# Retraction Note: Effects of Novel Calpain Inhibitors in Transgenic Animal Model of Parkinson’s disease/dementia with Lewy bodies

**DOI:** 10.1038/s41598-026-47178-2

**Published:** 2026-04-14

**Authors:** Getaw Worku Hassen, Leo Kesner, Alfred Stracher, Abraham Shulman, Edward Rockenstein, Michael Mante, Anthony Adame, Cassia Overk, Robert A. Rissman, Eliezer Masliah

**Affiliations:** 1 Department of Emergency Medicine, New York Medical College, Metropolitan Hospital Center, New York, USA; 2 Center for Drug Delivery Research, SUNY Downstate Medical Center, New York, USA; 3 Department of Otorhinolaryngology, SUNY Downstate Medical Center, New York, USA; 4 Department of Neurosciences, University of California, San Diego, La Jolla, California 92093-0624 USA; 5 Division of Neurosciences and Laboratory of Neurogenetics, National Institute on Aging, National Institutes of Health, Bethesda, MD 20892 USA

Retraction of: *Scientific Reports* 10.1038/s41598-018-35729-1, published online 27 December 2018

The Editors have retracted this Article.

After publication, concerns were raised regarding unusual features in some of the data presented in the Figures, specifically:

• The blots in Fig. 4c appear to have highly similar features between different lanes;

• Some images in Fig. 5a appear to have repetitive patterns, which are unlikely to occur naturally in microscopy images.

The Editors therefore no longer have confidence in the presented data.

Elezier Masliah and Getaw Worku Hassen do not agree with this retraction. Leo Kesner, Alfred Stracher, Abraham Shulman and Edward Rockenstein are deceased. The other authors have not responded to any correspondence from the editor or publisher about this retraction.

